# Silver Nanomaterial-Immobilized Desalination Systems for Efficient Removal of Radioactive Iodine Species in Water

**DOI:** 10.3390/nano8090660

**Published:** 2018-08-26

**Authors:** Ha Eun Shim, Jung Eun Yang, Sun-Wook Jeong, Chang Heon Lee, Lee Song, Sajid Mushtaq, Dae Seong Choi, Yong Jun Choi, Jongho Jeon

**Affiliations:** 1 Advanced Radiation Technology Institute, Korea Atomic Energy Research Institute, Jeongeup 56212, Korea; she0805@kaeri.re.kr (H.E.S.); chlee9406@kaeri.re.kr (C.H.L.); songlee@kaeri.re.kr (L.S.); sajidqau101@yahoo.com (S.M.); dschoi@kaeri.re.kr (D.S.C.); 2 Department of Chemistry, Kyungpook National University, Daegu 41566, Korea; 3 Department of Chemical and Biomolecular Engineering, Korea Advanced Institute of Science and Technology, Daejeon 34141, Korea; jung-e@kaist.ac.kr; 4 School of Environmental Engineering, University of Seoul, Seoul 02504, Korea; jeongsunwook@gmail.com; 5 Radiation Biotechnology and Applied Radioisotope Science, University of Science and Technology, Daejeon 34113, Korea

**Keywords:** bioremediation, desalination, membrane, nanocomposite, radioactive iodine, silver nanomaterials

## Abstract

Increasing concerns regarding the adverse effects of radioactive iodine waste have inspired the development of a highly efficient and sustainable desalination process for the treatment of radioactive iodine-contaminated water. Because of the high affinity of silver towards iodine species, silver nanoparticles immobilized on a cellulose acetate membrane (Ag-CAM) and biogenic silver nanoparticles containing the radiation-resistant bacterium *Deinococcus radiodurans* (Ag-DR) were developed and investigated for desalination performance in removing radioactive iodines from water. A simple filtration of radioactive iodine using Ag-CAM under continuous in-flow conditions (approximately 1.5 mL/s) provided an excellent removal efficiency (>99%) as well as iodide anion-selectivity. In the bioremediation study, the radioactive iodine was rapidly captured by Ag-DR in the presence of high concentration of competing anions in a short time. The results from both procedures can be visualized by using single-photon emission computed tomography (SPECT) scanning. This work presents a promising desalination method for the removal of radioactive iodine and a practical application model for remediating radioelement-contaminated waters.

## 1. Introduction

In recent decades, radioactive isotopes have widely been used for industrial and medical applications that have introduced drastic quantities of radioactive toxic pollutants to the environment [1,2]. Among many radioactive isotopes, large amounts of after-use radioactive iodine species (iodines) have been discarded following applications in radiation therapies and biomedical studies [3,4,5,6,7,8]. Moreover, the recalcitrant characteristics of exposed radioactive materials contribute to serious adverse effects such as acute diseases, metabolic imbalances, and genetic mutations [9,10,11,12,13,14]. Thus, the development of sustainable treatment methods for the removal of radioactive iodines is necessary for public health and environmental safety.

Many engineered nanomaterials have been used as adsorbents in nuclear waste treatment because of their large surface area. In addition, these materials possess high reactivity towards specific radioactive elements without additional chelating molecules [15]. Among them, silver metal-based materials have widely been used in the desalination of radioactive iodine wastes because of the high affinity of silver towards iodine species [16,17,18,19,20,21,22,23,24,25,26,27,28,29,30]. In a typical desalination procedure, these adsorbents should immerse in contaminated water for the removal of radioactive iodine, and thus radioactive elements-containing solid wastes generated by this process need to be separated from water [31]. Therefore, time-consuming processes such as centrifugation was required to harvest unsettled silver nanoparticles (AgNPs) and reprocess radioelement-contaminated solid adsorbents. Moreover, nano- or microscale silver materials are aggregated easily under high salt concentrations, causing physicochemical property losses [32,33].

Together, these observations inspired the design of a more efficient and stable silver particle-based desalination method for the removal of radioactive iodine waste in two ways by the incarceration of silver-based adsorbents. First, AgNPs were immobilized on a cellulose acetate membrane (CAM) and evaluated for desalination performance in a continuous-flow system. If radioisotopes are existed in homogeneous aqueous media, the engineered membrane can easily be applied to the separation step. However, when significant slurry or insoluble materials are contained in liquid contaminants, membrane-based equipment is unsuitable for purification procedures. In such cases, the bioremediation could be considered for an alternative process, as this method has some advantages over the membrane-based method, including: (1) possibility for on-site remediation; (2) removal of complexed radioelement contaminants by simple genetic engineering; and (3) easily scaled remediation processing. Therefore, as a second method, we report biogenic AgNP-containing radiation-resistant bacterial cells as efficient adsorbent carriers for use in a novel bioremediation platform.

## 2. Materials and Methods

### 2.1. General Methods

Silver nitrate, sodium borohydride, trisodium citrate, and sodium iodide (non-radioactive) were purchased from Sigma-Aldrich Korea (Yongin, Republic of Korea). CAMs (pore size = 0.20 μm, diameter = 25 mm) were purchased from Advantec MFS. [^125^I]NaI was supplied by New Korea Industrial Co., Ltd. (Daejeon, Republic of Korea). The radioactivity of ^125^I was measured by using a radioactivity dose calibrator (Capintec, Inc., Florham Park, NJ, USA) and a radio-thin-layer chromatography (TLC) scanner (Bioscan, AR-2000, Poway, CA, USA). The amount of radioactivity was determined by using a γ-counter (PerkinElmer, 2480 Automatic γ-counter, Waltham, MA, USA). Single-photon emission computed tomography/computed tomography (SPECT/CT) images were obtained by using an Inveon SPECT/CT system (Siemens, Erlangen, Germany). Silver nanomaterials on the CAMs and the surfaces of bacteria were observed using a field-emission scanning electron microscope (FE-SEM, Inspect F50, FEI, Mahwah, NJ, USA) under high-performance conditions with accelerating voltages reaching 15 kV. The elemental composition of the silver nanomaterials was analyzed by SEM-energy-dispersive X-ray (EDX) (EDAX Apollo XL, AMETEK, Mahwah, NJ, USA) analysis with accelerating voltages reaching 20 kV. EDX spectra were recorded in area scanning mode by focusing the electron beam onto a region of the sample surface. 

### 2.2. Synthesis of Citrate-Stabilized AgNPs

AgNPs with the average diameter of 30 nm were synthesized as described in the previous report [34]. In brief, solutions of sodium borohydride (NaBH_4_, 2.08 mM) and trisodium citrate (TSC, Na_3_C_6_H_5_O_7_, 2.08 mM) were mixed and the resulting solution was heated to 60 °C in the dark for 30 min. After heating, 2 mL of silver nitrate (AgNO_3_, 1.17 mM) was added dropwise and then the temperature was raised to 90 °C. When the temperature reached 90 °C, the pH was adjusted to 10.5 by adding 0.1 M NaOH solution. The resulting solution was then heated for 20 min. After the suspension was cooled to room temperature, the concentration of AgNPs was measured by using ultraviolet-visible (UV/vis) spectrometry (concentration = 7.0 × 10^−10^ M with an extinction coefficient of 1.45 × 10^10^ at the peak of 406 nm with a 1-cm path length). The solution was stored at 4 °C. The mean diameter of citrate-stabilized AgNPs was approximately 30 nm [35].

### 2.3. Preparation of the Ag-CAM

The commercially available CAM filter (pore size = 0.2 μm, diameter = 25 mm) was washed with 10 mL deionized water using a syringe. Next, 5 mL of citrate-stabilized AgNPs (7.0 × 10^−10^ M), was passed through the filter using a syringe at the rate of 1 mL s^−1^. The membrane filter was then washed with pure water three times, yielding a yellowish-brown colored filter. This Ag-CAM filter was kept under ambient conditions until testing in the desalination experiment. To analyze the surface of Ag-CAM by SEM-EDX, the composite membrane was isolated from the filter unit. 

### 2.4. Desalination of Radioactive Iodine Using Ag-CAM Filter Unit under Continuous In-Flow Conditions

To evaluate the efficiency of the Ag-CAM filter unit under continuous-flow conditions, [^125^I]NaI (3.7 MBq) was diluted with 50 mL aqueous media (pure water, 1.0 M NaCl, 1× phosphate-buffered saline (1× PBS), 10 mM NaI). Each aqueous radioactive iodine solution was then passed through an Ag-CAM at an in-flow rate of approximately 1.5 mL s^−1^ by using a syringe pump or by hand using lead gloves. The amount of residual radioactivity in the filtrate was measured by using a γ-counter.

The removal efficiency (%) was defined by the following equation to assess the adsorption capability of the Ag-CAM towards radioactive iodine:

Removal efficiency (%) = (*C*_0_ − *C*_e_)/*C*_0_ × 100 (1)

where *C*_0_ and *C*_e_ represent the initial (before filtration) and equilibrium (after filtration) concentrations of radioactive iodine, respectively.

The distribution coefficient (*K*_d_) was determined using the following equation:

(2) $$K_{d}=(C_{0}-C_{e})/C_{e}\times \frac{V}{M}$$

where *C*_0_ and *C*_e_ represent the initial and final concentrations of radioactive iodine, respectively; *V* denotes the volume of radioactive iodine solution (50 mL); and *M* is the mass of the adsorbent (0.1 mg AgNPs).

### 2.5. Preparation of Silver Nanomaterial-Containing *Deinococcus radiodurans* R1

Biogenic AgNPs were obtained using *Deinococcus radiodurans* cells as previously described [36,37]. In brief, *D. radiodurans* strain ATCC13939 was inoculated in a tryptone glucose yeast extract (TGY) liquid medium until the sample reached the optical density at 600 nm (OD_600_) of 1.0. After cultivation, silver nitrate was added to the cell cultures adjusted to the final concentration of 2.5 mM and incubated for 24 h at 30 °C. The cultures were centrifuged at 4000 rpm for 30 min, and the resulting pellets were washed three times with deionized water. The pellets were then re-suspended in 5 mL deionized water and used for further analysis.

The absorption spectrum of AgNP-embedded *D. radiodurans* (Ag-DR) was monitored by a UV/vis spectrophotometer (Epoch Microplate Spectrophotometer, BioTek Instruments, Daejeon, Republic of Korea) from 400 to 800 nm. The dynamic light scattering (DLS) analysis of the biogenic AgNPs was performed as described previously [34]. For analysis by SEM-EDX, the samples were fixed with 2.5% glutaraldehyde solution and then dehydrated with 30%, 50%, 70%, 80%, 90%, 95%, and 100% ethanol (EtOH). After dehydration, samples were freeze-dried overnight. The prepared samples were subjected to FE-SEM after platinum coating using ion sputtering for 1 min and the morphology and existence of AgNPs were observed.

### 2.6. Remediation Procedure of Radioactive Iodine Using Ag-DR

[^125^I]NaI solution (3.7 MBq) was added to the Ag-DR suspension in aqueous media (water or 1× PBS). The mixture was shaken on an orbital shaker. At each time point of 1, 5, and 15 min, an aliquot (0.5 μL) was withdrawn from the Ag-DR solution and spotted onto a silica-coated thin-layer chromatography (TLC) plate. The TLC plate was then developed using acetone as the mobile phase. After the solvent traveled to the top of the plate, the TLC plate was cut in half. The radioactivity of each piece was measured by using a γ-counter. The retention factor (*R*_f_) values of free ^125^I^−^ in solution and ^125^I^−^ in Ag-DR were 0.8 and 0, respectively. Therefore, the removal efficiency (%) was defined by the following equation to evaluate the desalination performance of Ag-DR:

Removal efficiency (%) = *C*_0_/(*C*_0_ + *C*_I_) × 100 (3)

where *C*_0_ and *C*_I_ represent the amounts of radioactivity at the bottom (*R*_f_ = 0) and higher position (*R*_f_ = 0.8), respectively.

### 2.7. SPECT/CT Imaging of Radioactive Iodine Captured by Ag-DR

Radioactive iodine ([^125^I]NaI, 3.7 MBq) was diluted with 50 mL of pure water. An aqueous radioactive iodine solution was then passed through the CAM or Ag-CAM. After the filtration procedure, the radioactivity in the membrane filter was imaged by SPECT/CT scanning.

Radioactive iodine ([^125^I]NaI, 3.7 MBq) was added to both Ag-DR and a wild *D. radiodurans* sample; each solution was shaken at room temperature for 15 min. Some of the cells were then transferred to a 1.5-mL tube and centrifuged for spinning down of the cells. Molecular imaging was performed by SPECT/CT.

## 3. Results and Discussion

### 3.1. Preparation of Ag-CAM

**Figure 1:**
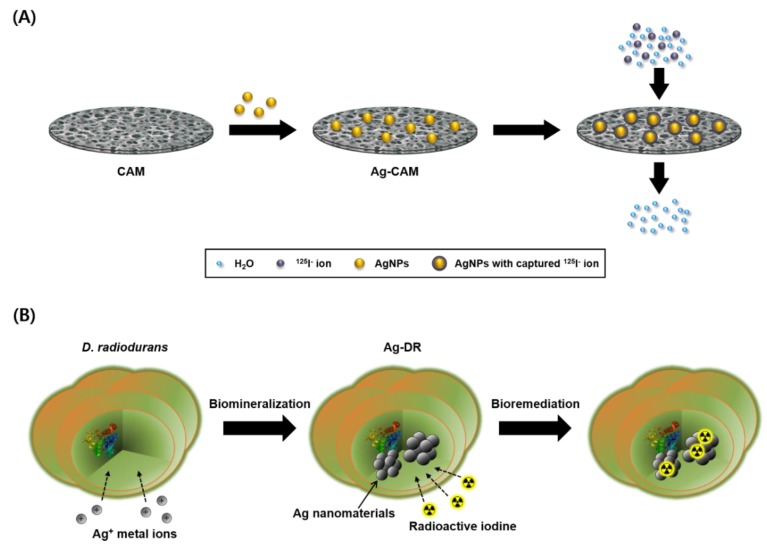
(**A**) Desalination of radioactive iodine by using Ag-CAM; and (**B**) bioremediation procedure of radioactive iodine anions using Ag-DR.

**Figure 2:**
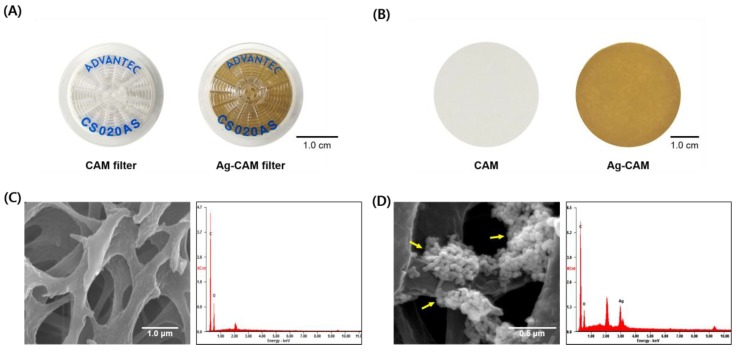
Characterization of Ag-CAM: (**A**) Photographic images of the CAM filter (left) and Ag-CAM (right) prepared using a syringe filter; (**B**) photographic images of the CAM (left) and Ag-CAM (right) prepared using a vacuum filter holder; (**C**) SEM–EDX analysis of the CAM (40,000×); and (**D**) Ag-CAM (100,000×). Yellow arrows in the images indicate AgNPs on cellulose fibers.

The main strategy for the desalination of radioactive iodines using silver nanomaterials is described in Figure 1. To examine the removal efficiency of radioactive iodine under continuous-flow conditions, the Ag-CAM filter is fabricated (Figure 2A,B). The immobilization of AgNPs on the CAM yields a homogeneous yellowish-brown color. The AgNPs incorporated in the filter unit were sustained stably without aggregate formation or elution from the membrane by continual washing with high-concentration salt solutions such as 1.0 M NaCl. Notably, Ag-CAM could be stored for several weeks without loss of stability or desalination performance. The hydroxyl and carbonyl groups in the polymeric cellulose acetate apparently efficiently stabilize the novel metal nanoparticles on the membrane [38,39]. SEM analysis of the surfaces of the Ag-CAM and CAM show that the nanomaterials are incorporated stably on the cellulose nanofibrils (Figure 2C,D). Elemental analysis of the membrane using EDX spectroscopy showed a set of peaks representing silver, along with carbon and oxygen atoms from the carbohydrate units in the cellulose polymer. These analyses verify the successful preparation of the composite membrane. The adsorption capacity of iodide anions on the Ag-CAM filter with a surface area of 4.91 cm^2^ was measured as approximately 31 mg of I^−^/g of AgNPs.

### 3.2. Desalination of Radioactive Iodine Using Ag-CAM

**Figure 3:**
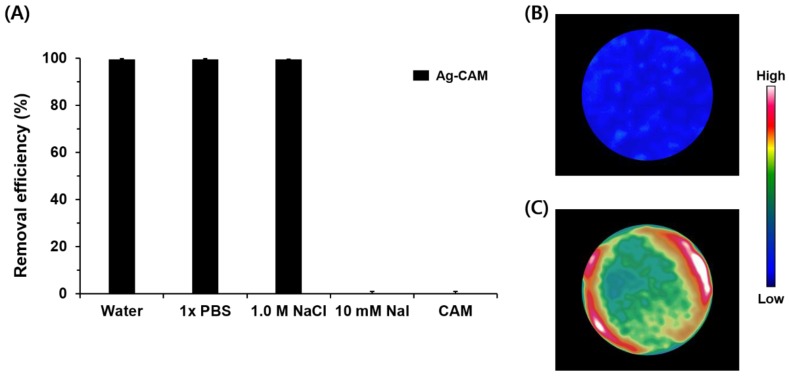
(**A**) Desalination of radioactive iodine using Ag-CAM in several aqueous solutions; (**B**) SPECT image of post-filtration non-modified CAM; and (**C**) SPECT image of post-filtration Ag-CAM.

The removal efficiency of Ag-CAM was investigated in the continuous-flow system. The filtration process was performed as shown in Figure 1A. The radioactive iodine solutions (typical concentration of 1.0 nM, 3.7 MBq/50 mL) were passed through the Ag-CAM at an in-flow rate of 1.5 mL s^−1^; the amount of radioactivity in the filtrate was then measured using a γ-counter. After a single filtration step, the concentration of ^125^I^−^ in pure water is dramatically decreased from 1 to 0.004 nM (Figure 3A). The removal efficiency reaches 99.6% and the distribution coefficient (*K*_d_) exceeds 10^6^ mL g^−1^. However, the unmodified CAM does not remove radioactive iodine under the same operation. The Ag-CAM maintained excellent removal efficiency under the presence of high concentrations of competing anions (1.0 M NaCl and 1× PBS). An excellent removal efficiency (99.5%) is observed in 1.0 M NaCl solution, in which the ratio of Cl^−^ to ^125^I^−^ anions ([Cl^−^]:[^125^I^−^]) reached 10^9^:1. However, in the presence of excess non-radioactive iodine, most radioactive iodine passed though the column, because the AgNPs immobilized on the CAM are covered with ^127^I^−^ anions. The desalination results are further visualized by SPECT/CT scanning. After filtering the [^125^I]NaI solution, the radioactive species, initially detected in the aqueous solution, is efficiently captured by the nanocomposite membrane (Figure 3B). However, the CAM filter unit captures no iodide anions via the same operation (Figure 3C). Thus, these images also confirm the Ag-CAM-mediated removal of radioactive iodine in a continuous-flow aqueous system. The nanocomposite membrane used in this study offer a simpler and more practical method to efficiently capture radioactive species from various aqueous solutions. By a simple filtration process, the amount of radioactive iodine in water is reduced significantly, with a removal efficiency of ≥99.5% in the presence of competing anions. In addition, the desalination of 50 mL of aqueous solution can be accomplished in 1 min. These results compare favorably with our previous report, which applied gold nanoparticles as adsorbents for radioactive iodine [40]. In the previous study, approximately 1.6 mg of gold was necessary to prepare the desalination membrane filter [41]; the present method uses approximately 0.1 mg AgNPs to achieve high desalination performance. Because silver is much cheaper than gold, the proposed Ag-CAM is more practical for the treatment of radioactive iodine wastes.

### 3.3. Remediation of Radioactive Iodine Using Ag-DR

In general, the most important feature of a water treatment system is its direct applicability to a polluted environment. Although silver metal-based adsorbents show high removal efficiencies, they have physical and spatial limitations for on-site remediation [36]. Meanwhile, bioremediation has certain advantages over physicochemical methods, including cost-effectiveness, eco-friendliness, and practicality [42]. Thus, bioremediation is considered as an alternative to previous physicochemical treatment processes. However, no reports on the application of biogenic silver nanoparticles on the desalination of radioactive iodine wastes exist. This may be because of the high radiation dose emitted by radioactive iodine. Thus, treatment of radioactive iodine wastes using AgNPs immobilized in the radiation-resistant extremophile *D. radiodurans* was investigated (Figure 1B).

**Figure 4:**
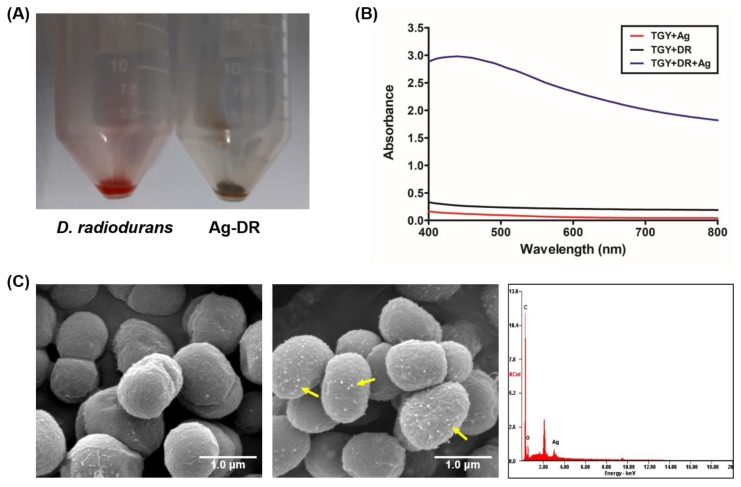
Characterization of Ag-DR: (**A**) Photographic image of *D. radiodurans* (left) and Ag-DR (right); (**B**) UV/vis spectra of *D. radiodurans* and Ag-DR; and (**C**) SEM image of *D. radiodurans* (left, 50,000×), Ag-DR (center, 50,000×), and EDX analysis of Ag-DR (right). Yellow arrows in the images indicate AgNPs on *D. radiodurans*.

During the culturing of *D. radiodurans* with 2.5 mM AgNO_3_, the color of the culture broth gradually changes from light orange to pale gray with increasing incubation time (Figure 4A). The UV/vis spectrum of the culture broth shows an absorption peak at 400–450 nm, indicating the formation of silver nanomaterials in the *D. radiodurans* strain (Figure 4B). The dynamic light scattering (DLS) analysis of nanomaterials isolated from *D. radiodurans* showed silver nanomaterials of approximately 30 nm in size. In addition, SEM-EDX analysis also clearly displays the silver nanomaterials synthesized by the bacterial cells (Figure 4C).

**Figure 5:**
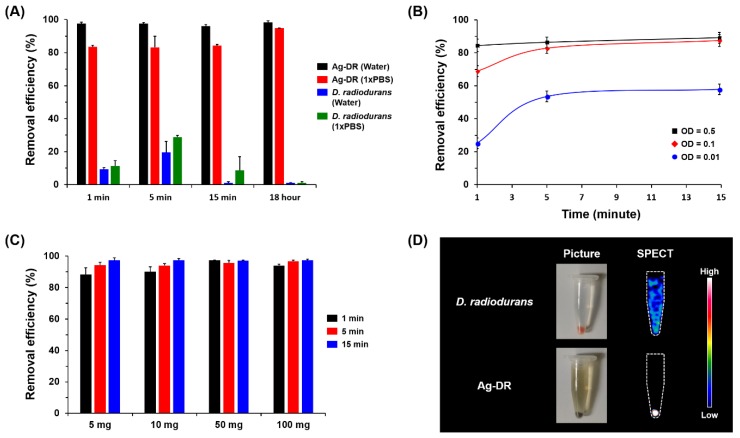
(**A**) Removal efficiency of Ag-DR in water and 1× PBS; (**B**) uptake kinetics for removal of radioiodine using smaller concentrations of Ag-DR in water for 15 min (OD = optical density at 600 nm); (**C**) removal efficiency of freeze-dried Ag-DR in water for 15 min; and **D**) photographic and SPECT/CT images of Ag-DR and *D. radiodurans* after ^125^I^−^ incubation.

Next, to investigate the desalination efficiency, radioactive iodine (3.7 MBq [^125^I]NaI) was added to Ag-DR and wild *D. radiodurans* (~10^9^ cells). The amount of radioactivity captured by the Ag-DR was then analyzed by a γ-counter and each experiment was performed in triplicate. As shown in Figure 5A, Ag-DR shows rapid uptake kinetics at the beginning of incubation; >97% radioactivity is captured by Ag-DR in 1 min. After overnight incubation (18 h), almost all iodine anions are stably retained in the Ag-DR, suggesting that Ag-DR provides efficient and sustainable remediation process. Meanwhile, *D. radiodurans* with no silver adsorbents show only non-specific retention of radioactive iodine. Although the remediation kinetics are slowed (84% in 15 min) in the presence of high concentrations of competing ions (1× PBS), the removal efficiency of 95% is obtained over a prolonged incubation time (18 h). Furthermore, the kinetics of the removal of radioactive iodine mostly depends on the amount of Ag-DR as can be seen in Figure 5B. However, a desalination efficiency of ~90% is obtained with a smaller amount of Ag-DR (0.1×) in a short time. We also investigated the removal capability of freeze-dried Ag-DR, considering the portability of the bacteria. Suspensions of 5, 10, 50, and 100 mg freeze-dried Ag-DR were mixed with equal amounts of radioactive iodine solution. As shown in Figure 5C, faster adsorption kinetics occurs with higher bacterial cell levels. After 15 min, satisfactory removal efficiency (>97%) is obtained with 5 mg freeze-dried Ag-DR.

To further confirm the bioremediation process, a molecular imaging study was performed using SPECT imaging. Radioactive iodine (3.7 MBq) was added to both Ag-DR and wild *D. radiodurans* and incubated for 15 min at room temperature. As shown in Figure 5D, the SPECT imaging analysis shows a strong radioactive signal at the bottom of the Ag-DR sample, exactly overlapping with photograph of the Ag-DR. However, *D. radiodurans* alone does not capture ^125^I^−^ ions; thus, most of the radioactivity retains in the supernatant. These observations clearly demonstrate that the AgNP-containing bacterial cells successfully capture the radioactive ^125^I^−^ ions. Although the Ag-DR shows good desalination performances, more successful examples of microbial bioremediation processes can be expected through guided strain development integrated with bioprocess engineering and systematic biotechnology.

## 4. Conclusions

In the present study, we developed a desalination process for the removal of radioactive iodine from water. First, an Ag-CAM filter was constructed and tested regarding its removal efficiency of radioactive iodide ions (^125^I^−^). It showed the removal efficiency and distribution coefficient (*K*_d_) of >99.6% and 10^6^ mL g^−1^, respectively. Next, biogenic AgNP-containing *D. radiodurans* was developed as a platform strain for on-site bioremediation. Approximately 3.7 MBq of radioactive iodine was successfully captured by the biogenic AgNPs immobilized in the cells within 15 min. Consequently, it is expected that the immobilized silver nanomaterials-based desalination method will provide a promising system worth to investigate large-scale nuclear waste management.
